# Comparative analysis of RNA enrichment methods for preparation of *Cryptococcus neoformans* RNA sequencing libraries

**DOI:** 10.1093/g3journal/jkab301

**Published:** 2021-08-26

**Authors:** Calla L Telzrow, Paul J Zwack, Shannon Esher Righi, Fred S Dietrich, Cliburn Chan, Kouros Owzar, J Andrew Alspaugh, Joshua A Granek

**Affiliations:** Department of Medicine, Duke University School of Medicine, Durham, NC 27710, USA; Department of Molecular Genetics and Microbiology, Duke University School of Medicine, Durham, NC 27710, USA; Department of Biology, Duke University, Durham, NC 27710, USA; Department of Microbiology and Immunology, Tulane University School of Medicine, New Orleans, LA 70112, USA; Department of Molecular Genetics and Microbiology, Duke University School of Medicine, Durham, NC 27710, USA; Department of Biostatistics and Bioinformatics, Duke University Medical Center, Durham, NC 27710, USA; Department of Biostatistics and Bioinformatics, Duke University Medical Center, Durham, NC 27710, USA; Duke Cancer Institute, Duke University, Durham, NC 27710, USA; Department of Medicine, Duke University School of Medicine, Durham, NC 27710, USA; Department of Molecular Genetics and Microbiology, Duke University School of Medicine, Durham, NC 27710, USA; Department of Biostatistics and Bioinformatics, Duke University Medical Center, Durham, NC 27710, USA; Duke Cancer Institute, Duke University, Durham, NC 27710, USA

**Keywords:** RNA sequencing, RNA enrichment, ribosomal RNA, non-coding RNA

## Abstract

RNA sequencing (RNA-Seq) experiments focused on gene expression involve removal of ribosomal RNA (rRNA) because it is the major RNA constituent of cells. This process, called RNA enrichment, is done primarily to reduce cost: without rRNA removal, deeper sequencing must be performed to compensate for the sequencing reads wasted on rRNA. The ideal RNA enrichment method removes all rRNA without affecting other RNA in the sample. We tested the performance of three RNA enrichment methods on RNA isolated from *Cryptococcus neoformans*, a fungal pathogen of humans. We find that the RNase H depletion method is more efficient in depleting rRNA and more specific in recapitulating non-rRNA levels present in unenriched controls than the commonly-used Poly(A) isolation method. The RNase H depletion method is also more effective than the Ribo-Zero depletion method as measured by rRNA depletion efficiency and recapitulation of protein-coding RNA levels present in unenriched controls, while the Ribo-Zero depletion method more closely recapitulates annotated non-coding RNA (ncRNA) levels. Finally, we leverage these data to accurately map the *C. neoformans* mitochondrial rRNA genes, and also demonstrate that RNA-Seq data generated with the RNase H and Ribo-Zero depletion methods can be used to explore novel *C. neoformans* long non-coding RNA genes.

## Introduction

RNA sequencing (RNA-Seq) is a powerful tool for quantifying gene expression in diverse organisms. Despite the rapid and continual decrease in sequencing costs, the expense of sequencing is often the limiting factor in designing RNA-Seq experiments. Due to this cost constraint, enrichment of the RNA classes of interest, hereafter referred to as “RNA enrichment,” is an important step in library preparation for most RNA-Seq experiments. Ribosomal RNA (rRNA) is the most abundant RNA, generally constituting more than 90% of the total RNA in a cell (Giannoukos *et al.* 2012). Despite this, rRNA is rarely of interest in RNA-Seq experiments because its main function is as a component of ribosomes. Therefore, 90% or more of the data is useless when generated without RNA enrichment. RNA enrichment aims to reduce the content of rRNA in the library, eliminating sequencing capacity wasted on uninformative data and reducing the cost of data storage and analysis, thus decreasing the overall cost of the experiment.

There are many different methods for RNA enrichment and many products available based on these different methods. When selecting an RNA enrichment method and product there are two key considerations: (1) the fraction of rRNA removed and (2) the side effects on other RNA in the sample. RNA enrichment methods either specifically target the RNA of interest, most commonly mRNA, for isolation or specifically target rRNA for removal (Zhao *et al.* 2014). The most common mRNA isolation method for eukaryotes, Poly(A) isolation, uses an oligo(dT) affinity matrix. Raw RNA is hybridized to the matrix, which preferentially binds the 3′ polyadenylation sequence of mRNA. By enriching polyadenylated mRNA, rRNA, which lacks 3′ polyadenylation, is depleted *de facto*. Although mRNA isolation methods are typically efficient in eliminating rRNA, they fail to capture any RNA molecules lacking polyadenylation, such as non-coding RNA (ncRNA). They are also only applicable to eukaryotes, since mRNA in prokaryotes is generally not polyadenylated. Most rRNA removal methods involve hybridization of sequence-specific probes to rRNA. These probes target the rRNA for depletion. In the Ribo-Zero depletion method, the probes are synthesized with a molecular tag, which is used to bind the probe-rRNA complex to beads, allowing the complexed rRNA to be removed from solution (Zhao *et al.* 2014). In the ribonuclease H (RNase H) depletion method, sequence-specific DNA probes hybridized to rRNA target the rRNA for enzymatic degradation by RNase H, which specifically degrades RNA from RNA-DNA complexes (Morlan *et al.* 2012). The duplex-specific nuclease (DSN) method indiscriminately depletes high abundance sequences by denaturing and reannealing the prepared RNA-Seq library, then treating with a DSN to degrade all double-stranded DNA. Under the conditions used for reannealing, high abundance sequences are much more likely to find a complementary sequence, so high abundance sequences, including but not limited to rRNA, are preferentially removed from the pool (Yi *et al.* 2011).

The Poly(A) isolation and DSN methods are attractive because they are broadly applicable without any organism-specific adaptation: the Poly(A) method works in all eukaryotes and the DSN method should work in any organism. However, the rRNA removal methods (Ribo-Zero and RNase H) are more targeted and are therefore expected to have fewer side-effects on biologically important RNA molecules, such as protein-coding RNA and ncRNA. The downside inherent in the targeted nature of the rRNA depletion methods is that the sequence-specific probes must be designed for the organism under experimentation or a close relative for maximal efficacy. Because rRNA is the most highly conserved sequence across the tree of life (Isenbarger *et al.* 2008), probes designed for an evolutionarily distant species will often work, but efficiency of rRNA depletion decreases with evolutionary distance. For all rRNA depletion methods, the performance of these rRNA removal methods can vary by organism, so it is important to assess them on the organism of interest.

The budding yeast *Cryptococcus neoformans* is a human fungal pathogen that infects more than 200,000 people annually and causes excessive mortality among immunocompromised patient populations, such as those with HIV/AIDS and those receiving immunosuppressive cancer therapies (Rajasingham *et al.* 2017). Research on *C. neoformans* helps us better understand this pathogen, contributes to the development of treatments for *C. neoformans* infections, and advances our understanding of fungal pathogens in general. RNA-Seq has been used extensively in *C. neoformans* studies to elucidate regulatory networks of protein-coding genes and mRNA structure and function (Chang *et al.* 2014; Chen *et al.* 2014; Janbon *et al.* 2014; Gish *et al.* 2016; Chow *et al.* 2017; Brown *et al.* 2018, 2020; Burke *et al.* 2018; Wallace *et al.* 2020; Yu *et al.* 2020). While there is intense interest in the role of ncRNA in higher eukaryotes such as humans, relatively little work has explored the implications of ncRNA in fungi, with focus largely on model fungi such as *Saccharomyces cerevisiae* (Bird *et al.* 2006; Bumgarner *et al.* 2009, 2011; Gelfand *et al.* 2011; Parker *et al.* 2018) and *Schizosaccharomyces pombe* (Ding *et al.* 2012; Atkinson *et al.* 2018). However, multiple recent studies have demonstrated the importance of ncRNA in *Cryptococcus* biology and virulence, including microRNA (miRNA) (Jiang *et al.* 2012; Liu *et al.* 2020), small interfering RNA (siRNA) (Janbon *et al.* 2010; Wang *et al.* 2010; Liu *et al.* 2020), and long non-coding RNA (lncRNA) (Fan *et al.* 2005; Chacko *et al.* 2015; Liu *et al.* 2020).

In planning and analyzing RNA-Seq experiments in *C. neoformans*, it is essential to understand the side-effect profile of the RNA enrichment method used. RNA enrichment methods that alter levels of RNA of interest may give misleading or incorrect results; this is a special concern for analysis of ncRNA. Here, we assess three different enrichment methods for RNA-Seq applications in *C. neoformans*: RNase H depletion, Ribo-Zero depletion, and Poly(A) isolation. The Ribo-Zero depletion (“Ribo-Zero Kit Species Compatibility Tables”; Trevijano-Contador *et al.* 2018; Liu *et al.* 2020) and Poly(A) isolation (Bloom *et al.* 2019; Brown *et al.* 2020) methods have been used previously in *C. neoformans*, while the RNase H method has not. However, none of these methods have been evaluated in *C. neoformans* in comparison to each other, much less to unenriched controls. By performing this controlled experiment, we quantified the efficiency of rRNA depletion and determined the side-effects of each depletion method on non-rRNA genes.

We find that the RNase H depletion method is more efficient than the Ribo-Zero depletion and the Poly(A) isolation methods in removing rRNA. Additionally, we report that the RNase H depletion method is highly specific. It more closely reflects protein-coding RNA levels present in unenriched controls than the other two methods, and reflects annotated ncRNA levels present in unenriched controls nearly as well as the Ribo-Zero depletion method. Because the RNase H and Ribo-Zero depletion methods both retain ncRNA, we leveraged these unique datasets to demonstrate the feasibility of identifying novel *C. neoformans* lncRNA when rRNA removal methods are utilized. Collectively, this work demonstrates that RNase H depletion is an effective RNA enrichment method for use in preparation of *C. neoformans* RNA-Seq libraries, further emphasizes the role of RNA enrichment in design of economical RNA-Seq experiments, and highlights the importance of knowing the side-effect profile when choosing an RNA enrichment method.

## Materials and methods

### Strains, media, and growth conditions

The *C. neoformans* var. *grubii* H99 (*MAT*α) wild-type strain was used for all experiments. This strain was maintained on yeast extract-peptone-dextrose (YPD) medium (1% yeast extract, 2% peptone, 2% dextrose, and 2% agar for solid medium).

### RNA-Seq library preparation

Three biological replicate samples (A, B, and C) were used for all analyses. Samples were prepared by growing H99 to mid-logarithmic growth phase in three separate flasks of liquid YPD medium, with 150 rpm shaking. Approximately 1 × 10^9^ cells from each sample were pelleted, resuspended in fresh YPD medium, and incubated at 30°C for 90 min with 150 rpm shaking. Cells were then pelleted, flash frozen on dry ice, and lyophilized for ∼18 h. Total RNA was isolated using the Qiagen RNeasy Plant Mini Kit (Qiagen, Valencia, CA, USA); on-column DNase digestion was performed to ensure elimination of contaminating genomic DNA. Total RNA quantity and quality were assessed using the Agilent 2100 Bioanalyzer. Purified total RNA was subsequently stored at −80°C. Aliquots from each total RNA sample were treated with one of three different RNA enrichment methods: the RNase H method for selective depletion of rRNA (Morlan *et al.* 2012; Adiconis *et al.* 2013), the Ribo-Zero rRNA Removal Kit (Yeast) (Illumina, San Diego, CA, USA), and the NEBNext^®^ Poly(A) mRNA Magnetic Isolation Module (NEB #E7490) (New England Biolabs, Ipswich, MA, USA). RNA-Seq libraries were prepared from these enriched samples and from unenriched control samples (*i.e.*, “Unenriched”) using the NEBNext^®^ Ultra™ II Directional RNA Library Prep with Sample Purification Beads (NEB #E7765) and NEBNext^®^ Multiplex Oligos for Illumina^®^ (Dual Index Primers Set 1) (NEB #E7600) (New England Biolabs, Ipswich, MA, USA). Libraries were pooled and sequenced by the Duke Sequencing and Genomic Technologies Shared Resource on an Illumina NextSeq 500 using the High-Output Kit to produce 75-basepair single-end reads.

It should be noted that while all work was done with the same three total RNA samples, the enrichment, library preparation, and sequencing were done in two batches, approximately one year apart. In between, total RNA samples were stored at −80°C. Ribo-Zero-treated, Poly(A)-treated, and Unenriched RNA control libraries were prepared and sequenced in the first batch. RNase H-treated, replicate Poly(A)-treated, and replicate Unenriched RNA control libraries were prepared and sequenced in the second batch. All Unenriched RNA samples were compared and shown to be highly correlated (Figures 2A–4A), demonstrating that batch effect and differences in total RNA storage times did not confound comparisons. It should also be noted that the Ribo-Zero-treated libraries, the Poly(A)-treated libraries, and the Unenriched libraries were prepared by multiple individuals, which may explain some of the sample variation within those groups, while the RNase H-treated libraries were all prepared by a single individual. The individual who prepared each library is noted in the library metadata deposited at GEO.

### RNase H depletion

The RNase H depletion method (Morlan *et al.* 2012; Adiconis *et al.* 2013) is described briefly here; a more detailed protocol is included in Supplementary File S1. The hybridization reaction mixture consisted of 1 µg of total RNA, 1 µl of 5× Hybridization Buffer (1000 mM NaCl, 500 mM Tris-HCl, pH 7.5), 0.65 µl of 100 µM pooled targeting oligos (discussed below), and nuclease-free water to bring the reaction to 5 µl. Oligo hybridization was performed in a thermocycler with the following program: 2 min at 95°C, ramp from 95°C to 22°C at −0.1°C/s, 5 min at 22°C. After hybridization, samples were transferred to ice and RNase H (New England Biolabs, Ipswich, MA, USA) was added: 2 µl RNase H (5 U/µl), 1 µl 10× RNase H Reaction Buffer, 2 µl nuclease-free water. RNase H digestion was performed at 37°C for 30 min. After RNase H digestion, samples were stored on ice while adding DNase I (New England Biolabs, Ipswich, MA, USA): 4 µl DNase I (2 U/µl), 10 µl 10× DNase I Reaction Buffer, 76 µl nuclease-free water. DNase I digestion was performed at 37°C for 30 min. After DNase I digestion, samples were transferred to ice. rRNA depleted RNA was purified from the reaction mixture with the Zymo RNA Clean & Concentrator-5 kit (Zymo Research, Irvine, CA, USA) according to manufacturer instructions and eluted in 12 µl nuclease-free water. Finally, 5 µl of the eluted RNA was input to the library prep using NEBNext^®^ Ultra II Directional RNA Library Prep Kit for Illumina (New England Biolabs, Ipswich, MA, USA).

### Analysis overview

All genomic analyses used genome build CNA3 of H99 *C.* *neoformans* var. *grubii* (accession GCA_000149245.3). The genome sequence and annotation were downloaded from release 39 of the Ensembl Fungi database (Kersey *et al.* 2016). For mapping mitochondrial rRNA genes, the original GTF downloaded from Ensembl Fungi was used. For all subsequent analyses, a modified GTF was used which included the newly mapped mitochondrial rRNA genes.

Analysis was performed using scripts written in the R programming language, Bash, and publicly available software detailed below. Custom R scripts used the following R and Bioconductor packages: biomartr, Biostrings, BSgenome, callr, CoverageView, cowplot, DESeq2, dplyr, foreach, fs, genomation, GenomicAlignments, GenomicFeatures, GEOquery, ggbio, ggplot2, ggpubr, gridExtra, Gviz, here, knitr, magrittr, matrixStats, plyr, purrr, readr, rentrez, rmarkdown, Rsamtools, rstatix, rtracklayer, R.utils, stringr, tibble, tidyr, tools, and utils.

### Mapping of mitochondrial rRNA genes

Coverage depth was plotted for all reads mapped to the mitochondrial chromosome for data generated from the first batch of Unenriched libraries. Visual inspection of these plots clearly indicated two regions with coverage depth several orders of magnitude higher than the rest of the chromosome. These regions do not overlap with any annotated feature in the mitochondrial chromosome. We determined the boundaries of these regions, extracted the sequences of the putative rRNA genes, and confirmed by BLASTn (Altschul *et al.* 1990) that these regions were homologous to known fungal mitochondrial small (positions 16948-18316) and large (positions 6710-9326) subunit rRNA genes (Supplementary Figure S1). A modified version of the *C. neoformans* genome annotation supplemented with our mitochondrial rRNA gene annotations is included (Supplementary File S2).

### Design of rRNA targeting oligonucleotides

Short DNA oligos were designed to target all nuclear rRNA genes (CNAG_10500, CNAG_10501, CNAG_10502, CNAG_10503) and the newly annotated 15S and 21S rRNA mitochondrial genes. In order to guide degradation of all rRNA by RNase H, the DNA oligos must be complementary to the rRNA and completely tile the rRNA. For simplicity and cost minimization, the design goal for rRNA targeting oligos was for them to be 50 nucleotides in length with no gaps between adjacent oligos. For genes with lengths that were not multiples of 50 nucleotides, single nucleotide gaps were introduced between oligos to allow for end-to-end coverage. Two, 55 nucleotide oligos were used to tile CNAG_10503, which is 111 basepairs long. Oligos were validated by mapping them to the H99 genome and confirming that they tiled as expected and mapped to the antisense strand. This validation process identified several partial duplications of the mitochondrial rRNA, putative nuclear mitochondrial DNA (numts) (Hazkani-Covo *et al.* 2010), and nuclear rRNA. These duplications were found in CNAG_04124, CNAG_06164, CNAG_07466, CNAG_12145, CNAG_12438, CNAG_13073, and in the region between CNAG_10503 and CNAG_03595. CNAG_13073 was excluded from analysis of rRNA depletion specificity because the rRNA duplication it contains is in an exon and on the sense strand, meaning that reads originating from rRNA genes can be misassigned to CNAG_13073. The other duplications do not result in spurious counts because they are either not in an exon or inserted antisense relative to the “host” gene.

The code used to design oligos should be applicable to other genomes; it is located within the file generate_rnaseh_oligos.Rmd, which is available, as described below, with the rest of the software developed for this project. This Rmarkdown document generates a TSV file in the correct format for pasting into the ordering template supplied by Eurofins; we have included a copy of the TSV generated for this project as a Supplementary File S3. The 179 oligos were ordered from Eurofins Genomics LLC at a 10 nmol synthesis scale, with salt-free purification, resuspended to 100 µM, and shipped on dry ice. Upon receipt, all oligos were thawed, pooled, aliquoted, and stored at −80°C. Total cost for oligos (not including shipping) was less than $1000. This provided over 21 ml of pooled oligos (179 oligos at 120 µl per oligo), enough for over 33,000 reactions. Therefore, while the upfront cost of oligos is substantial, the per reaction cost is about $0.03.

### Bioinformatics and statistical data analyses

Basic assessments of sequence data quality were performed using FastQC (Andrews 2010) and MultiQC (Ewels *et al.* 2016). Raw sequencing reads were trimmed and filtered using fastq-mcf (EA-Utils version 1.04.807) (Aronesty 2011) and adapter sequences were extracted from the manufacturer-provided “Sample Sheet NextSeq E7600” template for the NEBNext^®^ Multiplex Oligos for Illumina^®^ (Dual Index Primers Set 1) (https://www.neb.com/-/media/nebus/worksheet-samples/e7600_reverse-complement-workflow-sample-sheet.csv, accessed June 23, 2021). Reads were then mapped to the genome and read counts were generated using STAR (version 2.5.4b) (Dobin *et al.* 2013). For quantification of reads mapped to genes, we use the fourth column (“counts for the 2nd read strand aligned with RNA”) of the STAR ReadsPerGene.out.tab because the NEBNext^®^ Ultra™ II Directional RNA Library Prep uses the dUTP method for strand-specific library preparation. All sequencing was done on an Illumina NextSeq 500, which has a flow cell with four lanes that are fluidically linked (*i.e.*, one pool is simultaneously loaded onto all four lanes). While we expect there to be some lane effects, we expect these to be less than fluidically independent lanes. Because of this, and for simplicity, reads were combined across all four lanes for analysis of depletion efficiency and specificity.

### Analysis of rRNA depletion efficiency

To calculate the percentage of rRNA reads per library, Rsamtools (version 2.2.2) was used to extract reads from the STAR generated BAM files and determine the number of reads mapped to the rRNA genes. We did not use rRNA counts generated by STAR because STAR excludes multimapping reads from per gene read counts. As discussed above, several rRNA genes are partially duplicated elsewhere in the genome. Because STAR excludes multimapping reads from gene counts, it undercounts reads mapping to the rRNA genes that are partially duplicated. We confirmed the source of reads that mapped to rRNA duplicated regions by evaluating context: the count level of these reads corresponded to the level of expression of the rRNA genes from which the duplications seem to have arisen and not the level of expression of the genes (or genomic region in the case of the partial duplication of CNAG_10500) that seem to be the “acceptor sites” of these duplications. The percentage of total reads that mapped to rRNA genes was then calculated.

### Enrichment correlation analyses

Per gene read counts were generated by STAR as described above. Read counts for each library were combined across all four lanes using DESeq2::collapseReplicates, each library’s counts were normalized by its size factor, then an average count per gene was calculated for each enrichment method across all replicates. The mean normalized count of the Unenriched replicate libraries was considered the reference count for each gene. Specificity of each enrichment method was determined by calculating the Pearson correlation of the mean normalized count for each enrichment method with that of the Unenriched libraries. Variation among the Unenriched libraries was quantified by cross-correlation: Pearson correlation was calculated for each Unenriched replicate library with the normalized mean of the other five Unenriched replicate libraries. Scatterplots were generated to visualize the correlation of replicate enriched libraries with the Unenriched libraries. While calculation of mean normalized counts used all replicates for each method, scatterplots are only shown for one technical replicate of each RNA sample for each enrichment method (Supplementary Figures S2, S3, S4, and S6). In addition to analyses across all genes, calculation of Pearson correlation and generation of scatterplots was repeated for subsets of genes, as annotated for “gene_biotype”: protein-coding genes, ncRNA, and tRNA, according to each gene’s annotation. rRNA genes and CNAG_13073 were excluded from all correlation analyses and scatterplots.

To determine specifically which genes are “lost” by the Poly(A) isolation method, we identified genes with counts at least eight-fold lower in the Poly(A)-treated libraries than in the Unenriched libraries, after first excluding genes with very low expression in the Unenriched libraries (genes with less than 50 total read counts across all Unenriched libraries). These thresholds were chosen to identify obvious outliers in the Poly(A)-treated libraries and were confirmed by visual inspection of the identified genes (Supplementary Figure S6). We selected these thresholds to be more conservative than thresholds commonly used to identify genes with biologically relevant differences in expression (total reads of at least 10 and fold change of at least 2). To explore whether deadenylation explains the failure of the Poly(A) isolation method to capture a subset of these protein-coding transcripts, we visualized read depth across each of the corresponding genes and compared these distributions to read depth across a random set of control genes (Supplementary Figure S7). The control genes were selected from a subset that had total read counts in the same range as the “lost” protein-coding genes.

### LncRNA analysis

To demonstrate the feasibility of identifying novel lncRNA from RNA-Seq datasets generated using rRNA removal methods, we applied LncPipe (Zhao *et al.* 2018) to the data generated from the RNase H-treated, Ribo-Zero-treated, and Unenriched RNA libraries; Poly(A)-treated libraries were not included because they were expected to contain few, if any, reads derived from lncRNA. The published version of LncPipe only appears to work with data generated from human samples, so we forked the LncPipe repository and modified it to enable analysis of *C. neoformans* data. Details of the forked repository are provided below.

We developed an Rmarkdown document to perform all necessary pre-processing for running LncPipe (Zhao *et al.* 2018). This pre-processing involved automated reformatting of the input GTF file, preparing a subset of the GTF containing only protein-coding genes and another subset containing only non-protein-coding genes, generating a *C. neoformans* specific model for CPAT (one component of LncPipe), and generating a Bash script which itself runs LncPipe. LncPipe itself was run in Singularity with the bioinformatist/lncpipe Docker image built by the LncPipe developers (https://hub.docker.com/layers/bioinformatist/lncpipe/latest/images/sha256-9d97261556d0a3b243d4aa3eccf4d65e458037e31d9abb959f84b6fe54bb99a2?context=explore). Within LncPipe, STAR was used for mapping reads and the final step, LncPipeReporter, was not run.

## Results

### RNase H depletion is most efficient in removing rRNA

We focus the majority of our analyses on the RNase H depletion and Poly(A) isolation methods, because, of the three RNA enrichment methods assessed here, they are the two that are still available for use. To provide some context to our RNase H depletion method results, we also include analyses on the Ribo-Zero depletion method, which was frequently used in fungal RNA-Seq experiments before its discontinuation.

**Figure 1:**
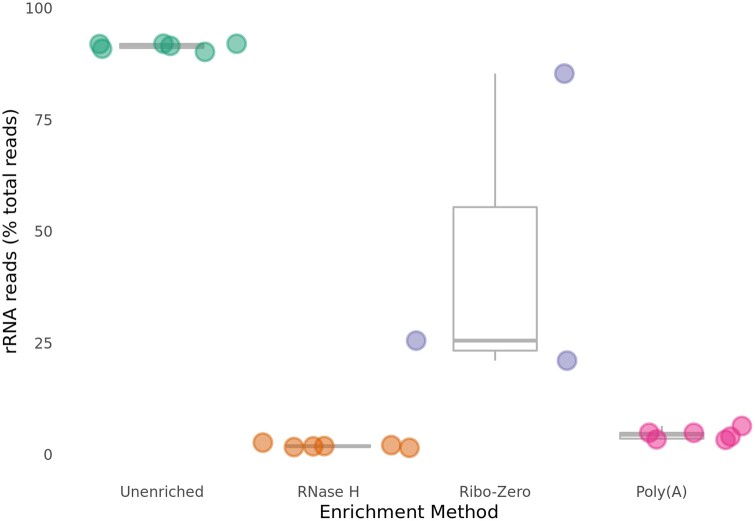
The RNase H depletion method is highly efficient in eliminating rRNA. The percentage of rRNA reads in each library is plotted. The RNase H depletion method has the most efficient depletion (lowest percentage of rRNA reads), with the Poly(A) isolation method a close second, and the Ribo-Zero depletion method a distant third. Unenriched libraries show that rRNA makes up most of the RNA in *C. neoformans*.

As an initial assessment, we evaluated the efficiency with which each enrichment method removed rRNA. To do so, we quantified the percentage of total reads that mapped to rRNA genes for each method and compared these percentages to those of Unenriched RNA control libraries generated by sequencing identical RNA samples without any enrichment. As expected, the vast majority (∼90–92%) of reads in the Unenriched RNA control libraries map to rRNA genes (Figure 1). Both the RNase H-treated libraries (∼1.5–2.5%) and the Poly(A)-treated libraries (∼3–5%) display a significant reduction in the percentage of reads mapping to rRNA genes (Figure 1). The Ribo-Zero depletion method was previously found to be efficient in depleting fungal rRNA and was used successfully in RNA-Seq applications for various fungi (Illumina; Trevijano-Contador *et al.* 2018; Liu *et al.* 2020). We similarly evaluated the Ribo-Zero depletion method and observed that the number of mapped rRNA reads is significantly higher in the Ribo-Zero-treated libraries (∼21–85%) than in the RNase H-treated and Poly(A)-treated libraries (Figure 1). Overall, both the RNase H depletion and Poly(A) isolation methods demonstrate robust efficiency in removing fungal rRNA, with the RNase H depletion method modestly outperforming the commonly-used Poly(A) isolation method.

### RNase H depletion more closely reflects Unenriched non-rRNA levels than Poly(A) isolation

**Figure 2:**
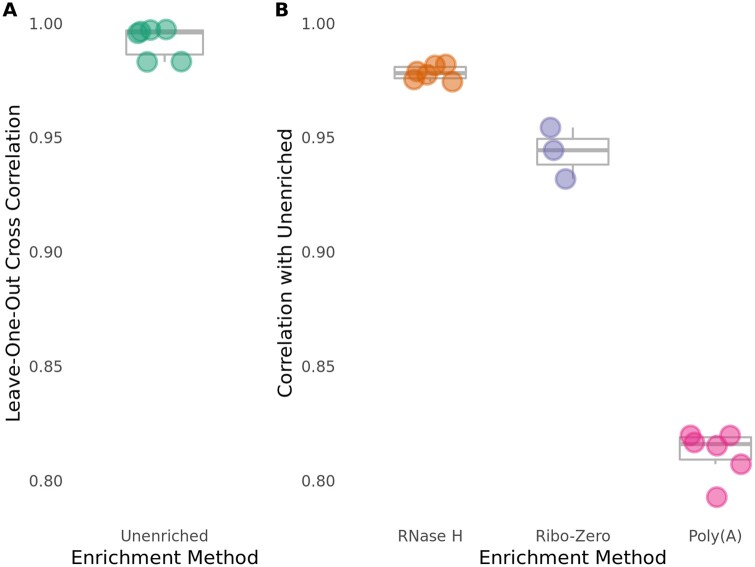
The RNase H depletion method is highly specific. Pearson correlations were calculated for normalized read counts of all annotated genes in the *C. neoformans* genome, excluding rRNA genes and genes containing coding-strand rRNA duplications. (A) Unenriched libraries have high internal consistency as determined by leave-one-out cross-correlation of each Unenriched library with the mean of other Unenriched libraries. (B) The RNase H depletion method has the best overall rRNA depletion specificity, as determined by Pearson correlation of read counts for all genes with the Unenriched libraries. Pearson correlation coefficient (R) was calculated between each enriched library and the gene-wise average of counts across all Unenriched libraries.

To compare the specificity of the three RNA enrichment methods, we determined the correlation between read counts in the enriched libraries to read counts in the Unenriched RNA control libraries generated from the same samples. To do so, we first calculated the correlation coefficient between normalized reads mapped to all non-rRNA genes from all Unenriched RNA samples, in order to determine the maximum achievable correlation between libraries. As expected, we observed that the Unenriched RNA samples are highly correlated (*R* = 0.983–0.997), demonstrating reproducibility between samples and batches (Figure 2A).

We compared the abilities of the RNase H depletion method and the Poly(A) isolation method to retain all non-rRNA following rRNA depletion. To do so, we calculated the correlation coefficient of normalized reads mapped to all non-rRNA genes in RNase H-treated andPoly(A)-treated libraries with normalized reads mapped to all non-rRNA genes from the Unenriched RNA control libraries. We found that the RNase H-treated libraries (*R* = 0.974–0.982) display a much better correlation with the Unenriched RNA control libraries than the Poly(A)-treated libraries (*R* = 0.793–0.820) for all reads mapping to non-rRNA genes (Figure 2B and Supplementary Figure S2). We similarly assessed the Ribo-Zero depletion method for preservation of all non-rRNA. Ribo-Zero-treated libraries (*R* = 0.932–0.954) display a much better correlation with the Unenriched libraries than the Poly(A)-treated libraries, but a slightly weaker correlation than the RNase H-treated libraries for reads mapping to all non-rRNA genes (Figure 2B and Supplementary Figure S2). This observation suggests that the RNase H depletion method may be more specific than the Poly(A) isolation and the Ribo-Zero depletion methods, in that it maintains non-rRNA levels observed in the Unenriched RNA control libraries.

### RNase H depletion more closely reflects Unenriched protein-coding RNA levels than Poly(A) isolation

**Figure 3:**
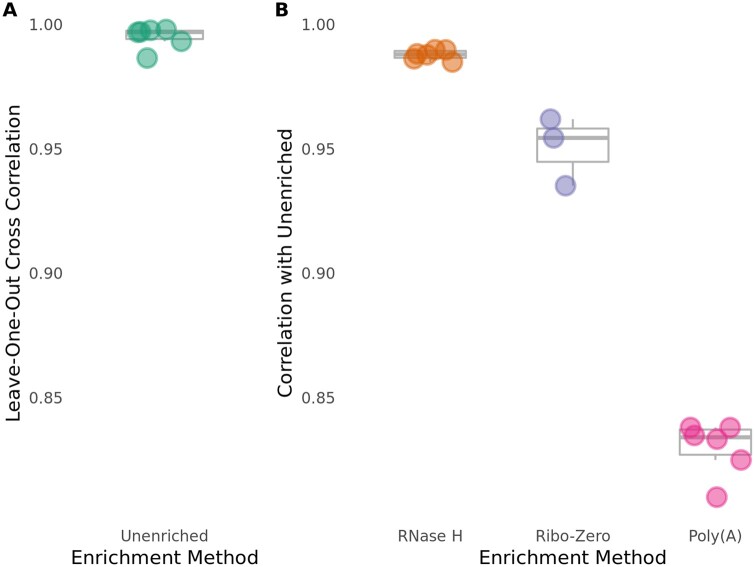
The RNase H depletion method is highly specific with respect to protein-coding genes. Pearson correlations were calculated in the same way as Figure 2, but only for protein-coding genes, excluding genes containing coding-strand rRNA duplications. (A) Unenriched libraries have high internal consistency for protein-coding genes. (B) The RNase H depletion method has the best rRNA depletion specificity for protein-coding genes.

We next assessed the ability of each RNA enrichment method to retain protein-coding RNA specifically. To do so, we calculated the correlation coefficient between normalized reads mapped to protein-coding genes from all Unenriched RNA samples, in order to determine the maximum achievable correlation between libraries. As expected, we observed that the Unenriched RNA samples are highly correlated (*R* = 0.986–0.998), demonstrating reproducibility between samples and batches (Figure 3A).

We compared the ability of the RNase H depletion method and the Poly(A) isolation method to retain protein-coding RNA following rRNA depletion. To do so, we calculated the correlation coefficient of normalized reads mapped to protein-coding genes in RNase H-treated and Poly(A)-treated libraries with normalized reads mapped to protein-coding genes from the Unenriched RNA control libraries. We found that the RNase H-treated libraries (*R* = 0.985–0.990) display a much better correlation with the Unenriched RNA control libraries than the Poly(A)-treated libraries (*R* = 0.810–0.838) for all reads mapping to protein-coding genes (Figure 3B and Supplementary Figure S3). We similarly assessed the Ribo-Zero depletion method for preservation of protein-coding RNA. Ribo-Zero-treated libraries (*R* = 0.935–0.962) display a better correlation with the Unenriched libraries than the Poly(A)-treated libraries, but a slightly weaker correlation than the RNase H-treated libraries for reads mapping to protein-coding genes (Figure 3B and Supplementary Figure S3). This observation demonstrates that the RNase H depletion method is more specific than both the Poly(A) isolation and Ribo-Zero depletion methods in maintaining protein-coding RNA levels observed in the Unenriched RNA control libraries.

### RNase H depletion more closely reflects Unenriched annotated ncRNA levels than Poly(A) isolation, but slightly less closely than Ribo-Zero depletion

**Figure 4:**
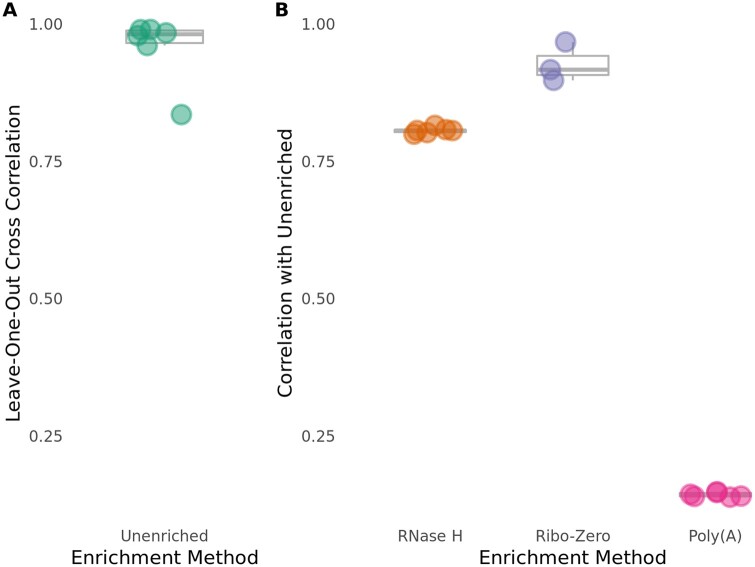
The RNase H depletion method is highly specific with respect to annotated ncRNA genes, although less so than the Ribo-Zero depletion method. Pearson correlations were calculated in the same way as Figure 2, but only for annotated ncRNA genes, excluding rRNA genes. (A) Unenriched libraries have high internal consistency for annotated ncRNA genes. (B) The Ribo-Zero depletion method has the best rRNA depletion specificity for annotated ncRNA genes.

A major advantage of using rRNA removal methods in RNA-Seq applications is their ability to retain ncRNA. We next assessed the ability of each RNA enrichment method to retain annotated ncRNA specifically. To do so, we calculated the correlation coefficient between normalized reads mapped to annotated ncRNA genes from all Unenriched RNA samples, in order to determine the maximum achievable correlation between libraries. Again, we observed that the Unenriched RNA samples are highly correlated (*R* = 0.835–0.990), demonstrating reproducibility between samples and batches (Figure 4A).

We compared the ability of the RNase H depletion method and the Poly(A) isolation method to retain annotated ncRNA following rRNA depletion. We calculated the correlation coefficient of normalized reads mapped to annotated ncRNA genes in RNase H-treated and Poly(A)-treated libraries with normalized reads mapped to annotated ncRNA genes from the Unenriched RNA control libraries. As expected, we found that the RNase H-treated libraries (*R* = 0.799–0.815) display a much better correlation with the Unenriched RNA control libraries than the Poly(A)-treated libraries (*R* = 0.139–0.149) for all reads mapping to annotated ncRNA genes (Figure 4B and Supplementary Figure S4). This result was expected because the Poly(A) isolation method specifically enriches RNA with polyadenylation and excludes all other non-polyadenylated RNA, including ncRNA and tRNA. One key advantage of methods that specifically remove rRNA, such as the RNase H depletion and the Ribo-Zero depletion methods, is that they “ignore” all RNA that is not specifically targeted for removal. As a result, these non-polyadenylated RNA species should maintain similar levels as the input Unenriched RNA.

As a better assessment of the ability of the RNase H depletion method to retain annotated ncRNA, we compared it to the Ribo-Zero depletion method. Ribo-Zero-treated libraries (*R* = 0.897–0.967) display a slightly better correlation with the Unenriched libraries than the RNase H-treated libraries for reads mapping to annotated ncRNA genes (Figure 4B and Supplementary Figure S4). The higher correlation of Ribo-Zero-treated libraries with Unenriched libraries seems to be driven by CNAG_12993, the annotated ncRNA gene with the highest counts in the Unenriched libraries, but much lower counts in the RNase H libraries. When CNAG_12993 is removed from analysis, the RNase H and Ribo-Zero depletion methods perform similarly (Supplementary Figure S5; RNase H *R* = 0.936–0.952, Ribo-Zero *R* = 0.892–0.959). There is no clear explanation for the poor performance of the RNase H depletion method with CNAG_12993.

We also explored the ability of each enrichment method to retain tRNA. Fewer than 10 reads mapped to each tRNA gene in all libraries, likely due to size selection in the library preparation, precluding any meaningful analysis (data not shown).

### Data generated from RNase H- and Ribo-Zero-treated libraries can be used to explore novel lncRNA

Because our correlation analyses demonstrated that both the RNase H and the Ribo-Zero depletion methods retain annotated ncRNA, we explored the feasibility of harnessing these datasets to identify novel lncRNA. We used an existing pipeline, LncPipe (Zhao *et al.* 2018), that was developed for a subset of model organisms, and modified it for application to *C. neoformans*. We applied this modified LncPipe pipeline to identify predicted lncRNA within our RNase H depletion, Ribo-Zero depletion, and Unenriched RNA datasets. Our lncRNA discovery analysis identified 11 predicted lncRNA within the *C. neoformans* transcriptome (Table 1). This analysis was performed to demonstrate an advantage of using rRNA removal methods like the RNase H and Ribo-Zero depletion methods for RNA-Seq experiments. These approaches provide investigators with the ability to explore novel lncRNA, which would otherwise be impossible using mRNA isolation methods like the Poly(A) isolation method.

**Table 1:** LncPipe identification of *C. neoformans* lncRNA

Name	Chromosome	Start	End	# Exons	Total exonic length	Mean TPM	Median TPM
LINC-CNAG_07358-1	1	996421	997387	2	863	6.689427833	6.2667
LINC-CNAG_07633-1	6	499352	499840	3	350	2.844233167	0
LINC-CNAG_07649-1	6	1351673	1352718	3	913	5.5161474	5.799715
LINC-CNAG_07769-5	9	828268	829327	3	919	9.2005976	10.76355
LINC-CNAG_07769-4	9	831951	833064	4	2019	5.333811583	5.026716
LINC-CNAG_07769-1	9	838389	840007	10	2730	4.443440783	3.846055
LINC-CNAG_04857-1	10	199988	203380	43	6849	12.5665295	12.729905
LINC-CNAG_04857-2	10	203693	205767	2	1983	1.903616517	1.67792
LINC-CNAG_01945-1	11	1333989	1334590	2	540	3.47618165	1.745465
LINC-CNAG_06521-2	13	743402	744570	4	1003	5.924291983	5.627535
LINC-CNAG_07042-1	13	750280	751009	3	609	5.950332833	4.02392

Predicted lncRNA were discovered by analysis of RNase H-treated, Ribo-Zero-treated, and Unenriched RNA libraries. The name (assigned by LncPipe), chromosomal location, exon number, exonic length, and transcripts per million (TPM) across samples are shown for all 11 lncRNA identified.

## Discussion

RNA enrichment is essential for cost-effectively generating data from an RNA-Seq experiment. We have demonstrated here that, in *C. neoformans* cells grown in permissive conditions, rRNA constitutes more than 90% of the total RNA; even higher percentages of rRNA have been observed in other species (Giannoukos *et al.* 2012). RNA-Seq experiments are typically aimed at quantifying protein-coding RNA, and increasingly also ncRNA. Efficient reduction of rRNA allows one to generate the desired sequencing depth of the RNA species of interest with one-tenth of the sequencing reads that would be required to generate the same depth from total, unenriched RNA.

To be effective, RNA enrichment methods must be efficient and specific. An efficient RNA enrichment method removes as much rRNA as possible. A specific RNA enrichment method does not affect other RNA species in the sample. We compared the rRNA removal efficiency of three commonly-used methods in *C. neoformans* samples. Application of the RNase H depletion method in *Cryptococcus* has, to our knowledge, never been reported. The Poly(A) isolation method (Bloom *et al.* 2019; Brown *et al.* 2020) and the now discontinued Ribo-Zero depletion method (Illumina; Trevijano-Contador *et al.* 2018; Liu *et al.* 2020) have both been used in RNA-Seq applications with *Cryptococcus* samples in the past. We find that both the untested RNase H depletion method, as well as the frequently used Poly(A) selection method, are very efficient in removing fungal rRNA. Surprisingly, the Ribo-Zero depletion method showed poor efficiency in *C. neoformans*, despite previous work showing efficient removal of various bacterial rRNA (Giannoukos *et al.* 2012). While the Ribo-Zero manufacturer predicted that the Ribo-Zero Yeast kit would work for *C. neoformans*, the probes were designed to target *S. cerevisiae*, which may explain the poor performance observed here. Of the three methods tested, the RNase H depletion method is the most efficient in removing fungal rRNA.

Following the removal of rRNA, the majority of remaining RNA is typically protein-coding. Ideally, the removal of rRNA should not have any effect on protein-coding RNA. In reality, there is no known method that can reduce rRNA without having some effect on non-target RNA, including protein-coding RNA. When assessing the ability of these RNA enrichment methods to retain protein-coding RNA, we observe that the RNase H depletion method is more specific than the Poly(A) isolation method and somewhat more specific than the Ribo-Zero depletion method, in that it more closely reflects protein-coding RNA levels observed in the Unenriched controls.

We evaluated the specificity of each RNA enrichment method in retaining annotated ncRNA. The Poly(A) isolation method is unable to retain ncRNA; this is as expected since it depends on 3′ polyadenylation, which is absent from ncRNA. The RNase H depletion and the Ribo-Zero depletion methods both perform well in retaining annotated ncRNA, with the Ribo-Zero depletion method being slightly more specific than the RNase H depletion method, in that it more closely reflects annotated ncRNA levels observed in the Unenriched controls.

To determine a possible mechanism impacting specificity of the Poly(A) isolation method, we identified the genes that are most underrepresented in the Poly(A)-treated libraries compared to the Unenriched libraries (Supplementary Figure S6). A total of 41 genes were identified as substantially underrepresented in the Poly(A)-treated libraries; as expected, 24 of these genes are non-coding genes (Supplementary Table S1). In analyzing the remaining 17 protein-coding genes that are underrepresented in the Poly(A)-treated libraries, we observed a pattern. The vast majority of these genes (12 of 17) are located on the mitochondrial chromosome (Supplementary Table S1). This observation is supported by previous work that has demonstrated that mitochondrial transcripts in fungi, including *Cryptococcus*, lack polyadenylation (Toffaletti *et al.* 2003; Chang and Tong 2012). In seeking to understand why the remaining five nuclear protein-coding genes are underrepresented, we visualized the read depth across them in the Unenriched libraries. We observed that reads are concentrated at the 5′ end and the middle of these genes, which suggests that a substantial fraction of the reads mapped to these genes arise from degradation intermediates (Supplementary Figure S7). Deadenylation is often a marker of mRNA degradation in eukaryotes, including fungi, so these underrepresented transcripts are likely degradation intermediates (Parker 2012). Because the Poly(A) isolation method depends on 3′ polyadenylation, it is not surprising that deadenylated degradation intermediates would be underrepresented in the Poly(A)-treated libraries. Work in other eukaryotic systems has suggested that deadenylated mRNA can have fates beyond degradation, including recycling to the translating pool of mRNA (Decker and Parker 2012). With this in mind, we conclude that the Poly(A) isolation method is highly effective in profiling mRNA encoded in the nuclear genome that is poised for canonical translation at a specific point in time. The RNase H depletion method, on the other hand, provides a more complete profile of all present mRNA, including functional mRNA, degradation intermediates, and mRNA that may not clearly fall into either category. Analyses such as those performed in Supplementary Figure S7 can be used to hypothesize about the functionality and fate of these transcripts. As a result, we find that the Poly(A) isolation method represents an overall accurate depiction of the true distribution of most protein-coding mRNA. However, the failure of the Poly(A) isolation method to efficiently capture RNA that is not polyadenylated makes it poorly suited for *C. neoformans* RNA-Seq experiments investigating mitochondrial mRNA, deadenylated mRNA, or ncRNA. As a result, experiments using the Poly(A) isolation method may overlook important biological phenomena, including the dynamics of mRNA with rapid turnover.

Interest in ncRNA has recently expanded in the fungal genetics field. The majority of work has focused on ncRNA in model systems (such as *S. cerevisiae*, *Neurospora crassa*, and *Aspergillus flavus*), in which lncRNA and natural antisense transcripts have been implicated in stress responses and development (Smith *et al.* 2008; Gelfand *et al.* 2011; Ding *et al.* 2012; Xue *et al.* 2014). Comparatively, little work has explored ncRNA in pathogenic fungi. For example, RNA interference is known to regulate transposon activity in *C. neoformans* (Janbon *et al.* 2010; Wang *et al.* 2010; Yadav *et al.* 2018). The first lncRNA in *Cryptococcus*, *RZE1*, was recently functionally characterized. *RZE1* is required for *Cryptococcus* yeast-to-hyphal transition and virulence through its regulation of the transcription factor *ZNF2* (Chacko *et al.* 2015). Additionally, siRNA, miRNA, and lncRNA are known to be secreted, albeit for an unknown purpose, by *C.* *neoformans* (Liu *et al.* 2020). We identified 11 predicted lncRNA in *C. neoformans* by mining our dataset by modifying LncPipe (Zhao *et al.* 2018) to run on genomic data from non-model organisms. It is important to note that this type of analysis is the initial step in identifying novel lncRNA; these predicted lncRNA must be experimentally validated using approaches such as northern blotting and ribosome profiling. These predicted lncRNA display reasonable expression across each respective gene, so they are interesting candidates for experimental validation and investigation for novel biological activity in conditions relevant to fungal pathogenesis, as many fungal ncRNA are induced in response to stressful stimuli (Supplementary Figure S8). Furthermore, the *C. neoformans* genome may contain undiscovered lncRNA that are not expressed in the permissive growth conditions used in these experiments but can be discovered using the approaches and analyses outlined here. Furthermore, it is possible that LncPipe requires optimization for identification of lncRNA in *C. neoformans*, because some lncRNA gene boundaries identified seem inconsistent with the reads, based on visual inspection (Supplementary Figure S8).

In conclusion, based on the data presented here, we consider the RNase H depletion method to be the most generally effective in preparation of *C. neoformans* RNA-Seq libraries. We also find that the Poly(A) isolation method is effective in many contexts. It does efficiently reduce rRNA reads (although not as efficiently as the RNase H depletion method), but it fails to capture biologically relevant RNA species that are not adenylated, including ncRNA, mRNA expressed in the mitochondria, and deadenylated mRNA. The RNase H depletion and the Ribo-Zero depletion methods both display strengths and weaknesses. The RNase H depletion method performs better in efficiency of rRNA reduction and specificity for protein-coding transcripts, while the Ribo-Zero depletion method performs moderately better in specificity for annotated ncRNA. While this work was being conducted, the Ribo-Zero product line was discontinued. It has since been replaced with the Illumina Ribo-Zero Plus rRNA Depletion Kit, which only targets human, mouse, rat, and bacterial rRNA. As a result, we conclude that the RNase H method may be the best option for RNA-Seq analysis of *C. neoformans*, as well as many other non-model organisms. While the RNase H depletion method has a substantial upfront cost to purchase DNA oligonucleotides (approximately $1000), we estimate that for this method our total cost per sample was less than $6.50 (more than half of this total was the final cleanup with the Zymo RNA Clean & Concentrator-5 kit).

## Data availability

The RNA-Seq data analyzed in this publication have been deposited in NCBI’s Gene Expression Omnibus (GEO) (Edgar *et al.* 2002; Barrett *et al*. 2013) and will be accessible through GEO Series accession number GSE160397 (https://www.ncbi.nlm.nih.gov/geo/query/acc.cgi?acc=GSE160397). The custom programs developed for processing and analyzing the RNA-Seq data are available in a GitHub repository (https://github.com/granek/rna_enrichment) and the version of the LncPipe pipeline that we modified to run on the H99 genome is available in a GitHub repository (https://github.com/granek/LncPipe) that was forked from the original. For purposes of reproducibility, all analyses were run within Singularity containers (v 3.5.2). All lncRNA discovery was performed using the bioinformatist/lncpipe Docker image (run within Singularity) provided by the LncPipe developers (https://hub.docker.com/layers/bioinformatist/lncpipe/latest/images/sha256-9d97261556d0a3b243d4aa3eccf4d65e458037e31d9abb959f84b6fe54bb99a2?context=explore). All other analyses were performed using a Singularity image which we built and is publicly available (library://granek/published/rna_enrichment). These resources include all programs, support files, and instructions for automatically replicating all analyses presented here using the data available from GEO.

All supplementary information is deposited in figshare: https://doi.org/10.25387/g3.15428664. Figure S1 contains a depth of coverage plot of the mitochondrial rRNA genes. Figures S2, S3, and S4 display scatterplot visualizations of rRNA depletion specificity summarized in Figures 2, 3, and 4, respectively. Figure S5 displays the rRNA depletion efficiency for ncRNA genes, excluding CNAG_12993. Figure S6 displays a scatterplot visualization of the genes that are underrepresented by the Poly(A) isolation method, Figure S7 displays the read depth across each of these genes, and Table S1 provides details of these genes. Figure S8 displays the read depth across each of the predicted lncRNA genes identified by LncPipe analysis. File S1 contains the RNase H depletion protocol. File S2 contains the Ensembl GTF with newly annotated mitochondrial rRNA. File S3 contains DNA oligonucleotide sequences used in the RNase H depletion method.
